# Transformative Integration: Navigating Opportunities and Challenges in a Medical School's Evolution Into Integrated Service Units

**DOI:** 10.7759/cureus.85631

**Published:** 2025-06-09

**Authors:** Chelsea Chang, Everardo Cobos, Michael D Sander, Beatriz Tapia, Michael Hocker

**Affiliations:** 1 Internal Medicine, University of Texas Rio Grande Valley School of Medicine, Edinburg, USA; 2 Orthopedic Surgery, University of Texas Rio Grande Valley School of Medicine, Edinburg, USA; 3 Faculty Affairs, University of Texas Rio Grande Valley School of Medicine, Edinburg, USA; 4 Surgery, University of Texas Rio Grande Valley School of Medicine, Edinburg, USA

**Keywords:** academic department, academic health centers, clinical service lines, integrated service unit, medical school structure

## Abstract

Medical schools must evolve with the changing healthcare landscape and financial pressures. Academic Health Centers have responded to these changes with a novel organizational model known as an integrated service line. Traditional medical school departments often create silos, lack of alignment, and financial burdens. To our knowledge, we were the first medical school to implement an integrated structure combining the tripartite missions of academics, research, and clinical services.

The University of Texas Rio Grande Valley School of Medicine accepted its charter class of 55 medical students in 2016 with a traditional medical school department model. By 2023, it had 154 full-time faculty, 14 departments, a growing clinical practice with 25 ambulatory sites, and no university teaching hospital. Facing changes in the local healthcare landscape, medical school leadership implemented a restructuring of the medical school and its outpatient health system into Integrated Service Units (ISUs) in 2023.

This study aims to (1) describe the institutional steps taken to implement an ISU model across academic, research, and clinical domains of a medical school; and (2) evaluate initial outcome observations in the first one-year post-implementation.

Over six months in 2023, the institution successfully implemented its vision of reorganizing the medical school across the academic, research, and clinical care areas. The new structure had seven ISUs: Primary and Community Care, Medicine and Oncology, Surgery Specialty and Musculoskeletal, Neuro and Behavioral Health, Surgery, Medical Education, and Clinical Support Services.

This paper delves into the challenges, opportunities, and key lessons learned. Embarking on the ISU transformation led us out of our comfort zones and beyond conventional paradigms.

Preliminary findings across academics, research, and clinical services are presented and support that within the first year, the ISU model has accelerated the ability to accomplish our vision of transforming the health of the Rio Grande Valley.

Next steps involve examining the long-term impact of the ISUs on the medical school, residencies, patients, and the health system. Areas of focus include financial success, faculty recruitment and retention, and research impact.

## Introduction

Medical schools have been structured with basic science and clinical departments for decades. These traditional medical school departments can contribute to silos, lack of alignment, and financial burden [1-3]. There has been a call for medical school departments to be dynamic [4]. Of note, the Association of American Medical Colleges (AAMC) in their 2024 report published that of the 156 medical schools surveyed, 22 medical schools changed their organizational models between 2019 and 2023 [5].

Over the last decade, Academic Health Centers (AHCs), which consist of a medical school, at least one other health professional school, and at least one affiliated or owned teaching hospital, have implemented what is termed “integrated service lines, integrated service units (ISUs), or integrated professional units (IPUs)” [6-9]. Even in these relatively new models, the medical school typically retains its traditional department structure [6]. As the cost of education and research rises and healthcare revenue declines [10], AHCs must have “unprecedented levels of alignment to preserve an environment that nurtures creativity” [9].

The AAMC hosted a webinar in 2021 titled “Academic Health Center of the Future-Organization Structure: Service Lines or Departments [11]?” ISUs have been shown to reduce cost, improve quality, and improve certain patient outcomes [2,7,8,12]. Morrice et al. present the design and analysis of an IPU defined as a “multi-disciplinary team of providers and staff who work together to cover the full care cycle for a given condition” [13]. Often, the ISUs or IPUs are introduced only for specific diseases, such as cancer care [14-17], emergency departments [8,18,19], musculoskeletal care [13,20,21], or heart and vascular service lines [2,22]. Johns Hopkins has published their “innovative and strategic organizational structure” with the Department of Pathology integrating clinical, education, and research programs [23].

Porter et al. recently published “Integrated practice units: a playbook for health care leaders,” which provides useful business perspectives [3], and Jain et al. also published recommendations to “deploy effective IPUs” [12]. A 2025 qualitative study was published on the role of leadership in IPUs, highlighting the importance of clear roles and a structured strategy for sharing information [24].

As of 2025, we did not find published reports in PubMed of medical schools with an ISU organizational structure throughout. To our knowledge, we are the first medical school to implement a school-wide integrated structure combining the tripartite missions of academics, research, and clinical services. This study aims to (1) describe the institutional steps taken to implement an ISU model across academic, research, and clinical domains of a medical school; and (2) evaluate initial outcome observations in the first one-year post-implementation.

## Materials and methods

The University of Texas Rio Grande Valley School of Medicine (UTRGV SOM) accepted its charter class of 55 medical students in 2016 and received full Liaison Committee on Medical Education (LCME) accreditation in 2023. By 2023, it had 154 full-time faculty composed of 43 PhD scientists, 86 physicians, 231 community faculty, 222 medical students, and 256 residents. The clinical practice arm has 25 ambulatory sites and no university hospital. It adhered to the conventional model of a medical school and had 14 departments, nine of which were clinical.

Operational challenges and departmental silos were hindering interprofessional collaboration and growth of the clinical practice and research areas. These challenges, in combination with several small departments with leadership vacancies and an increasingly competitive local healthcare market, spurred the Dean into action. The Dean and UTRGV’s President initiated the transformation of the ambulatory health system and the medical school into ISUs.

In June 2023, the executive team initiated “sensing meetings” with leaders representing the proposed ISUs, Human Resources (HR), and research and education deans. The meetings intentionally invited dissenting opinions and alternative solutions.

In July 2023, executive leadership collaborated with HR and the Office of Faculty Affairs to develop strategic communication and feedback mechanisms. The multifaceted approach included: (1) Small-group trainings in “Leading through Change” for newly appointed ISU Chairs; (2) Town Hall Meetings for faculty and staff; (3) Central online platform (Microsoft Teams Page) with FAQs, Communications and Organizational Charts; (4) Dedicated communication portal for ISU-related discourse.

Executive leadership designed these platforms to mitigate concerns, resolve queries promptly, and, crucially, cultivate a culture of trust and transparency. In August 2023, the Dean communicated widely that UTRGV SOM would transform into an ISU model with five stated aims: (1) strategically recruit, (2) grow research from bench to bedside, (3) expand class size, (4) build clinical specialty areas, and (5) drive informed resource decisions to achieve our mission.

**Figure 1:**
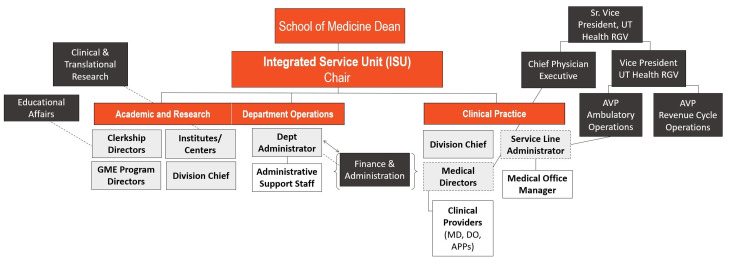
Sample Integrated Service Unit (ISU) structure and roles. The ISU structure shows key roles within the academic, research, and clinical realms, along with direct (solid line) and indirect (dotted line) reporting. Image credit: All authors. AVP, Assistant Vice President; MD, Doctor of Medicine; DO, Doctor of Osteopathic Medicine; APP, Advanced Practice Provider; RGV, Rio Grande Valley.

The Dean presented the new structure with a sample ISU shown in Figure 1. ISU leaders and HR ensured all faculty and staff had roles in the new structure with no Reduction in Force.

Approach to academics

Deans of Undergraduate and Graduate Medical Education evaluated the impact and regulations with their corresponding accrediting bodies. The LCME was notified of the organizational change and the intention to model interprofessional collaboration.

For the Graduate Medical Education programs, we assigned each residency program a primary ISU in which the faculty would have a primary appointment. The Accreditation Council for Graduate Medical Education was notified of the organizational change in our annual updates and of the intention to increase research, which had been a challenge for the residents and clinical faculty.

Approach to research

We integrated research institutes and departments within the most closely aligned ISU to enhance translational research. We considered establishing a standalone research ISU; however, we ultimately determined that such a structure would not sufficiently support the acceleration of bench-to-bedside discoveries. A central tenet of the ISU model, as aligned with our institutional goals, is the intentional integration of the three core missions - academics, research, and clinical care - within each unit. Creating a separate research ISU would risk reintroducing the very silos the model was designed to eliminate and would limit our ability to strategically align and synergize efforts across these domains.

Approach to clinical services

We designated each of the 25 ambulatory sites to a primary ISU in which its operations and financial transactions would occur. The larger multispecialty ISUs created divisions with division chiefs if there were more than five physicians of one specialty. Notably, the Medical Education ISU is the only unit that did not incorporate clinical services and has retained a predominantly PhD faculty focused on pre-clerkship education. 

Approach mapped to Kotter’s change model

In addition to the approach to the mission pillars as above, below is our approach mapped to a change model framework, Kotter’s eight-step organizational change model [25].

(1) Create a Sense of Urgency: Operational challenges, departmental silos, leadership vacancies, and a competitive healthcare market highlighted the need for immediate change and created a strong sense of urgency.

(2) Build a Guiding Coalition: Sensing meetings were conducted with leaders across departments, intentionally welcoming dissenting opinions and alternative solutions to build broad-based support.

(3) Form a Strategic Vision and Initiatives: The transformation into an Integrated Strategic Unit (ISU) model was announced, with five clearly defined goals: strategic recruitment, research growth, class size expansion, clinical specialty development, and informed resource allocation.

(4) Enlist a Volunteer Army: Newly appointed ISU Chairs and leaders received training in “Leading through Change.” Faculty and staff were actively engaged through Town Halls and other collaborative forums.

(5) Enable Action by Removing Barriers: Barriers were addressed through the implementation of communication platforms (Teams page, FAQs, portals) and reassurances that there would be no Reduction in Force, reducing resistance and fostering trust.

(6) Generate Short-Term Wins: Early wins included the rapid deployment of communication tools and the appointment of ISU leadership, providing clarity and building momentum.

(7) Sustain Acceleration: Momentum was maintained through continuous communication, ongoing feedback loops, and active support from Human Resources and the Office of Faculty Affairs.

(8) Institute Change: In August 2023, the Dean formally announced the transition to the ISU model. Contracts for the new structure were rolled out in September 2023, marking the institutionalization of the change.

## Results

Over six months in 2023, the vision for transforming the medical school organizational structure was implemented across academics, research, and clinical services. Preliminary findings across academics, research, and clinical services are presented below. Key lessons learned are in Table 1.

**Table 1:** Key lessons learned in integrated service unit implementation in a medical school.

Topic	Key lessons
Change Implementation	1. Engage local experts (Human Resources/Organizational Development, School of Business, Faculty Affairs) and implement a change model framework such as Kotter's model.
	2. Focus on articulating the necessity for change and inspiring teams to reimagine the medical school’s approach to fulfill its missions.
	3. Acknowledge change as a positive disruption to generate new ideas and opportunities.
	4. Define newly created roles and consider implementing a RACI (Responsible, Accountable, Consulted, and Informed) matrix [5].
Academic	1. Communicate organizational change and its benefits to accrediting bodies.
	2. Integrate research institutes in ISUs to increase research involvement with medical students, residents, and clinical faculty.
	3. Emphasize that ISUs reflect real-world healthcare scenarios, fostering interdisciplinary learning for students and residents.
Research	1. Integrate with clinical faculty with researchers to enhance the capacity for translational and clinical research.
	2. Ensure ISU Chairs, even if not researchers, listen and understand researchers and institutes to enhance their success.
	3. Focus on shared goals and identify new interdisciplinary opportunities.
Clinical Services	1. Chairs of multi-specialty ISUs should advocate and develop all faculty, not just within their specialty.
	2. Promote collaboration in the ISUs to enable quicker adaptation to healthcare changes.
Faculty Affairs	1. Develop a multifaceted approach for communication, fostering a culture of trust and transparency.
	2. Assess and explain any impact on promotion and tenure. There was no change in the criteria for promotion and tenure, as tenure was transferred to the newly formed units.
	3. Prepare for faculty identity crises. We retained specialties in titles (i.e., Assistant Professor of Internal Medicine) and nurtured identity by mentoring/teaching the next generation.

**Figure 2:**
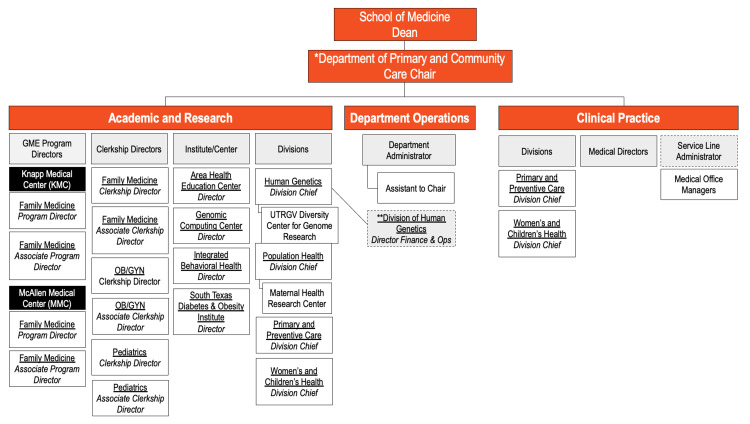
Primary and community care Integrated Service Unit (ISU) organizational chart. Image credit: All authors.

The new structure had seven ISUs: Primary and Community Care, Medicine and Oncology, Surgery Specialty and Musculoskeletal, Neuro and Behavioral Health, Surgery, Medical Education, and Clinical Support Services. The Primary and Community Care ISU chart is shown, for example, in Figure 2.

Outcomes in academics

Aligning academic curricula with the dynamic nature of ISUs proves complex. This challenge presents an opportunity to develop innovative curricula. ISUs provide a platform for collaborative learning across disciplines, fostering a holistic understanding of healthcare delivery. ISUs enable the creation of integrated educational programs reflecting real-world healthcare scenarios, better preparing students for the complexities of modern medical practice. For example, in the Primary and Community Care ISU, there was a newly formed Division of Women's and Children's Health, which included our pediatricians and obstetricians (Figure 2). Locating obstetrics and gynecology with pediatrics opens opportunities for medical students to see the continuum of infant-mother care.

The ISU structure expands clerkship learning opportunities and required clinical experiences by facilitating students to rotate through multispecialty teams, previously siloed in traditional departments. This provides an equivalent economy of scale without the need to proportionally increase faculty numbers. Efficiency is gained in ISU-level central support that would not have been feasible under the prior structure. One example from the Primary and Community Care ISU is the dedication of a full-time staff member to community faculty onboarding and engagement - a position that, given our size, would not have been feasible within a smaller, specialty-specific department or single clerkship.

At the same time, the ISU model presents challenges for academic faculty. The loss of same-specialty departmental leadership can lead to concerns about diminished professional identity and reduced visibility within the organization. Faculty have also expressed apprehension about the potential erosion of same-specialty mentorship opportunities, which are critical for career development, academic growth, and trainee guidance. Addressing these concerns will be essential to sustaining faculty engagement and ensuring the long-term success of the ISU model.

Outcomes in research

Groups that transitioned from departments to divisions or institutes within an ISU expressed concerns about potential loss of resources and diminished influence. Additionally, many ISU Department Administrators had limited experience managing research grants unanticipated knowledge gap given the centrality of grant funding in research-intensive units. ISU Chairs, all of whom were physicians, faced the challenge of building trust with research faculty and reassuring them that the restructuring was intended to support, not hinder, their success. In alignment with Kotter’s change model, generating short-term wins helped alleviate some concerns-for example, improved coordination with clinical sites facilitated participant enrollment in research studies.

Research integration has exposed more students, residents, and clinical faculty to the latest advancements in medical science. For example, immunology researchers and oncology clinicians have jointly published work and collaboratively worked on grant proposals. Clinical faculty now collaborate with our basic science faculty. They are facilitating discussion on how to do translational research to benefit our community. One promising example is the integration of the South Texas Center of Excellence for Cancer Research into the Medicine and Oncology ISU led to the successful receipt of an $18.4 million grant from the National Institutes of Health to establish the Rio Grande Valley Cancer Health Disparity Research Center in October 2024.

Outcomes in clinical services

The role of Chair expands in advocating for and developing all faculty, not just their specialty. For instance, the chair of the Surgery Specialty and MSK ISU could be an orthopedic surgeon, urologist, or otolaryngologist. Faculty expressed concern that multi-specialty ISUs could limit leadership and networking at the national level for leaders in these specialties. Some specialty societies have created a pathway acknowledging the varied organization of departments and permitted “appointed leaders” to join networking groups alongside chairs.

The interdisciplinary focus of ISUs promotes collaboration across disciplines. For example, in the Primary and Community Care ISU, family medicine physicians and internal medicine physicians have jointly explored and initiated participation in an Accountable Care Organization and have accelerated involvement in other value-based payment contracts.

ISUs offer flexibility in responding to evolving healthcare needs, enabling quicker adaptation to change in healthcare delivery. For example, during the leave of two pediatricians, family medicine physicians helped support the panel of patients on an interim basis. The multi-specialty ISUs were particularly helpful due to the small size of the ambulatory operations, with some specialties having only one to five physicians of that specialty. For our size practice, this significantly helped with shared resources, for example, Medical Assistants and other clinic staff could be more efficiently shared across disciplines within an ISU, which were previously siloed.

During sensing meetings and throughout the rollout, all were faced with the brutal facts of the changing healthcare landscape and an ineffective and unsustainable organizational structure. The ISU chairs are working closely with executive leadership to address challenging resource decisions to achieve the mission. In the first year, the ISU model resulted in a $3.5 million net savings with no Reduction in Force.

## Discussion

This report provides a critical reflection on the impact of the ISU school-wide one year after its implementation and aligns our findings with existing literature. Regarding our first aim, we successfully documented the institutional processes involved in implementing an ISU model across the academic, research, and clinical domains of a medical school. These steps have been contextualized within existing literature, providing a foundation for future implementation strategies. Furthermore, we systematically aligned our implementation with Kotter’s eight-step change model (1996), offering a structured framework for understanding our transformation process [25].

Arguably most significant is the adaptation of our organizational structure to support sustained change. As Kotter emphasized in his 2021 book, "the vast majority of organizations are struggling to adapt at a remotely adequate pace... The need to adapt is nothing new... What is new is how often we need to change, the pace at which we need to move, and the complexity and volatility of the context in which we are operating" [26]. This underscores the urgency and relevance of our structural adjustments within the dynamic landscape of medical education and healthcare delivery today.

Regarding our second aim, which was to evaluate initial observed outcomes within the first year post-implementation, the following section presents our reflections on the stated goals of the new organizational structure within existing literature.

Strategic recruitment

Our findings indicate that while ISUs are well-known in academic health centers (AHCs), their implementation in medical schools requires tailored recruitment strategies. This observation is consistent with research that emphasized the need for specialized recruitment approaches in academic settings [2,3]. As stated in the UMass Heart and Vascular Service Line Lessons Learned paper, "new recruits shared a commitment to the concept of an integrated Service Line, ensuring that the team could create an atmosphere conducive to new and nontraditional organizational and care delivery models" [2]. Our key lessons surrounding the importance of change implementation and communication skills are consistent with a qualitative study in four IPUs, looking specifically at the role of leaders in IPUs and recommending a structured strategy for sharing information [24].

Translational research growth

The collaboration between clinical and basic science faculty has fostered translational research. This aligns with the findings of Balser and Stead, who highlighted the importance of interdisciplinary collaboration in advancing clinical trials [9].

Expanding class size

The ISU structure's ability to expand learning opportunities without increasing faculty numbers reflects the efficiency gains reported by Phillips et al., who noted similar benefits in their study on their heart and vascular service line [2] and Sanfilippo et al. with the Johns Hopkins Department of Pathology integration [23].

Building clinical specialty areas

The cooperation among clinical services within the ISU structure has facilitated value-based payment contracts. This supports the findings of Keroack et al., who documented the benefits of integrated clinical services for achieving financial sustainability [7].

Informed resource decisions to achieve the mission

The ISU model has achieved significant net savings of $3.5 million, echoing results from others who reported cost savings in similar organizational or service line restructuring efforts [2,6]. Looking ahead, sustained success will depend on our ability to engage in value-based healthcare transformation identified in the *New England Journal of Medicine* (NEJM) IPU Playbook as "the central driver for organizations" [3]. While this shift remains aspirational within our current institution, it represents a critical next step in realizing the full potential of the ISU framework.

Limitations

This study has several limitations. First, it focuses on a single institution, which may limit the generalizability of its findings to other settings. Second, the evaluation captures only short-term outcomes of the transformation, highlighting the need for further longitudinal research to assess sustained impact. Additionally, the absence of quantitative outcome data limits the ability to measure the effectiveness of the change effort in objective terms. Future research could collect pre- and post-implementation data on ISUs-including metrics such as grant submissions, funded grants, MD/PhD collaborative projects, medical student publications, satisfaction levels among students, residents, and faculty, interdisciplinary course offerings, cost savings, and other relevant indicators provide more robust insight. Third, the Medical Education ISU is still in early development and lacks a clinical component, limiting assessment of clinical integration and value-based care outcomes. 

Another limitation is that the authors also served as key leaders in the organizational change effort. This dual role may introduce bias, potentially emphasizing success over challenges. However, it is common - and often necessary - for those directly involved in transformative change to document and analyze the process, given their unique access and contextual understanding. To mitigate this potential bias, the authors have aimed to present events transparently and to include diverse perspectives.

This study is primarily descriptive, intended to outline the steps taken and share preliminary observations.

## Conclusions

In conclusion, the implementation of the ISU model at UTRGV SOM has demonstrated significant improvements in collaboration, efficiency, and resource management across academics, research, and clinical services. Early outcomes indicate enhanced translational research, expanded educational opportunities, and cost savings, highlighting the model's potential for broader application in medical schools. However, further validation and long-term studies are necessary to assess the sustainability and adaptability of this approach across diverse institutions.
